# Ultrasonography Enthesitis and Synovitis Screening in Psoriatic Patients: A Case Control Study

**DOI:** 10.31138/mjr.180923.ues

**Published:** 2023-09-18

**Authors:** Soumaya Boussaid, Rania Ben Aissa, Sonia Rekik, Safa Rahmouni, Samia Jammali, Khaoula Zouaoui, Hela Sahli, Mohamed Elleuch

**Affiliations:** 1 Rheumatology Department, Rabta Hospital, Tunis, Tunisia,; 2 Faculty of Medicine of Tunis, University Tunis el Manar, Tunis, Tunisia,; 3 Research Unit LR 05 SP 01, La Rabta Hospital, Tunis, Tunisia

**Keywords:** psoriasis, psoriatic arthritis, ultrasonography

## Abstract

**Background:**

The clinical screening of enthesitis and synovitis in patients with psoriasis lacks specificity and sensitivity during the preclinical phase.

**Aims:**

to assess US subclinical synovitis and enthesitis in psoriatic patients compared with healthy controls.

**Methods:**

A cross-sectional study on 40 psoriatic patients and 40 healthy sex- and age-matched controls. US examination of 18 joints was performed along with 22 entheseal sites on the upper and lower limbs. US subscores were established according to the US abnormalities: inflammatory score (tendon thickening, hypoechogenicity, bursitis, Doppler signal), damage score (calcification, enthesophytes, bone erosion) and total score (the sum of inflammatory and damage scores).

**Results:**

US synovitis were more frequent in psoriatic patients (0.68%) than in controls (0.29%), but the difference was not significant. Patients with psoriasis had more US enthesitis (92,5%) compared to controls (40%)(p<0.001). The total number of enthesitis was higher in the psoriatic group (20.90%) compared to controls (4,78%)(p<0.001). There were more US abnormalities in the psoriatic group compared to controls for calcaneal tendon enthesis(p<0.001), distal patellar tendon enthesis(p<0.001) and deep flexor tendons of the finger enthesis(p<0.001). Compared to controls, psoriatic patients had a significantly higher inflammatory score (Mean±SD) (2.85±3.34 versus 0.58±1.17), damage score (3±2.57 versus 0.60±1.41), and total score (5.85±5.20 versus 1.18±2.07) (p < 0.001 each). Patients with scalp psoriasis had more US enthesitis (p=0.020).

**Conclusion:**

Our results indicate that US enthesitis and synovitis are more frequent in patients with psoriasis. Prospective studies with larger sample size are needed to define the contribution of US in predicting the clinical onset of PsA.

## HIGHLIGHTS

- Ultrasound enthesitis and synovitis are more frequent in patients with psoriasis.
- Patients with scalp psoriasis had more ultrasound enthesitis.
- There are more ultrasound abnormalities in the enthesis of the fingers deep flexor tendon in patients with psoriasis.

## INTRODUCTION

Psoriasis is a common chronic inflammatory skin disease affecting between 0.5 and 11.43% of the general population.^1^ Among psoriasis patients, up to 30% may develop Psoriatic arthritis (PsA).^2^ PsA could evolve into a destructive and disabling form,^3^ and its clinical aspects include skin, nails, enthesis, and joint involvement.^4^ Psoriasis precedes most often rheumatic manifestations by 7 to 12 years.^5^ The progression of inflammation from integument components to the synovial tissue and enthesitis during this interval is an opportunity to screen for subclinical lesions related to the development of PsA.^6^ The classification criteria for Psoriatic Arthritis (CASPAR)^7^ are based on clinical, biological, and radiographic criteria. It is the most used classification for PsA diagnosis, but its sensitivity in the detection of early PsA remains limited.^7–9^ Enthesitis is one of the major features of PsA. Clinical screening of enthesitis and synovitis remains difficult due to the proximity of the various joint structures that could be affected (known as the synovio-entheseal complex).^10–12^ Several clinical scores were developed for the evaluation of enthesitis in PsA but are still insufficient to detect enthesitis in comparison with imaging techniques.^13^ Standard radiography, ultrasound (US), and magnetic resonance imaging (MRI) are used for different stages of PsA evolution. Several studies in psoriasis patients without clinical articular manifestations suggest the role of US in screening for synovitis and/or enthesitis at a subclinical stage long before developing PsA.^12,14,15^ US detects more subclinical enthesitis (74%) than clinical examination (46%) and standard radiographs (26%).^16^

We conducted this study aiming to evaluate the prevalence of subclinical enthesitis and synovitis in psoriatic patients free from clinical arthritis or enthesitis compared with controls. Our second end point was to study the possible relationships of US findings with psoriasis characteristics such as phenotype, disease duration, severity, and current treatment.

## METHODS

### Study design

We conducted a monocentre case-control study between August 2020 and March 2021. The study was approved by the local Ethics Committee of the Hospital (approval CEBM.EPS.HR/30/2021). All participants signed an informed consent form.

### Study population

The study was conducted on patients with a diagnosis of psoriasis confirmed after clinical examination by a dermatologist and referred by the dermatology department. They were compared with 40 controls (without any inflammatory rheumatism or inflammatory pathology that can affect the entheses), sex and age-matched recruited from hospital workers and their relatives. The inclusion criteria were: age > 18 years and patients with psoriasis. The exclusion criteria were as follows: any history of inflammatory rheumatic, or crystal arthritis, fulfillment of the PsA CASPAR criteria, recent trauma, orthopedic surgery, amputation, or corticosteroid injection of the examined structures, or any biologic disease-modifying antirheumatic drugs (DMARDs) for psoriasis in the previous 3 months before the beginning of the study.

**Figure 1.**
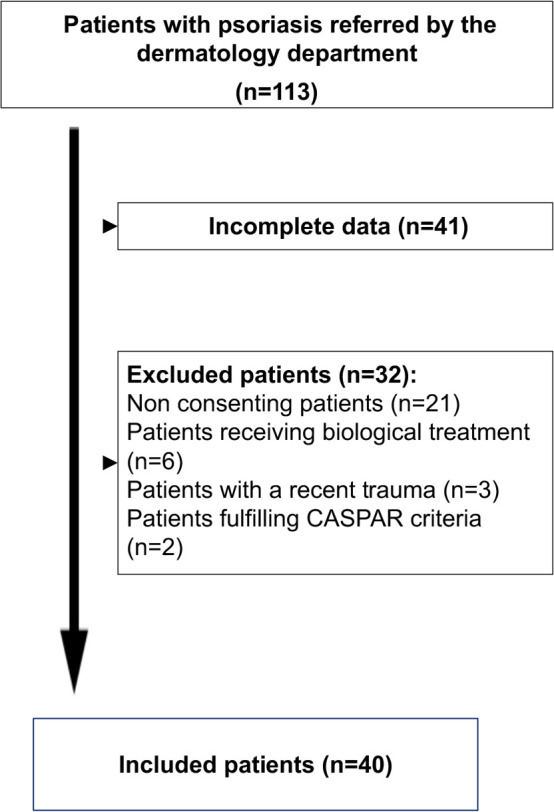
Flowchart of patient inclusion

To determine the sample size, we were based on a rate of synovitis and enthesitis in psoriatic patients of 3.2% and 11.6%, respectively,^17^ an error of 5%, and an accuracy of 7%. Thus, the number of subjects needed for a descriptive study would be 25 for synovitis and 40 for enthesitis. We selected 40 patients during the study period. Selection criteria are shown in **Figure 1.**

### Clinical evaluation and assessment

For each patient, we assessed: demographic characteristics, body mass index (BMI), current treatment for psoriasis, and sports activity within the last 2 weeks. We used the Arabic version of IPAQ-SF^18^ for all participants. We examined the joints using the 66/68 joint count for swelling (SJN) and tenderness (TJC).^19^ For the enthesis examination, we used two scores: the Leeds Enthesitis Index (LEI) and the Spondyloarthritis Research Consortium of Canada (SPARCC).^20,21^ Patients and controls were examined by a rheumatologist blinded to the US data.

The severity of psoriasis was assessed by a dermatolo-gist who scored the Body Surface Area (BSA) (severe > 10%, 3 % < moderate < 10%, < 3% mild), the psoriasis area severity index (PASI) score,^22^ and the Nail Psoriasis Severity Index (NAPSI)^23^ if a psoriasis nail involvement was documented.

Laboratory Measurement of C-reactive protein (CRP) was done using the enzyme-linked immunosorbent assay (ELISA) technique, which was considered positive if > 6 mg/l. The clinical and biological assessment was evaluated on the same day of the US examination.

### US protocol and acquisition

US examinations were performed at the Rheumatology Department of the Rabta Hospital using a Mylab Gamma (Esoate) equipped with a 6- to 18-MHz broadband linear transducer by an expert US rheumatologist in musculo-skeletal US imaging blinded of the clinical assessment findings. Grayscale (GSUS) and power Doppler (PDUS) at i) joints (bilateral wrist, metacarpophalangeal (MCP), proximal interphalangeal [PIP]), and ii) enthesis sites (deep digital flexor enthesis (from 2^nd^ to 5^th^ finger), lateral epicondyles, triceps tendon insertion into the olecranon tuberosity, proximal, and distal patellar tendons, calcaneal tendon, and plantar fascia) were performed.

Each joint was scanned in transverse and longitudinal planes. GSUS synovitis was defined as the presence of an abnormal hypoechoic intra-articular area with synovial effusion and/or synovial thickening, which may exhibit PDUS signals.

Enthesitis was defined according to the OMERACT when we found: abnormal hypoechoic tendon insertion, enthe-seal thickness of the tendon, bursitis, calcification, enthesophytes, bone erosion, or PDUS signals found within 2 mm of the bony cortex.^24^ The Madrid Sonographic Enthesitis Index (MASEI) score was used as a reference to the normal value of thickness of the lower limb and triceps insertion enthesis,^25^ for the common extensor tendon insertion on the lateral epicondyle, the cutoff established by Lee et al. was used.^26^

### US findings interpretation

US findings interpretations for both GSUS and PDUS synovitis were graded on a qualitative scale (0= absence, 1=presence).

Subscores for the US enthesis examination were elaborated:

- The damage score has been defined for structural abnormalities (calcification, enthesophytes, bone erosion)
- The inflammatory score has been defined for inflammatory abnormalities (tendon thickening, enthesis hypoechogenicity, bursitis, PDUS signals within 2 mm of the bony cortex).
- The total score was obtained by summing the damage and inflammatory score values.

Power Doppler settings were standardized with a Doppler frequency of 8–10 MHz and a pulse repetition frequency of 750 Hz. The color gain was adjusted just lower to the level that causes the appearance of noise artifacts

### Data analysis

The descriptive study included calculation of means±-standard deviation(SD), (minimum and maximum) and medians (interquartile range [IQR]) for quantitative variables with normal and nonnormal distribution, respectively; and absolute frequencies and percentages for categorical variables. Student’s t-test or Mann-Whitney test were used to compare independent means (eg, US scores, SJC, TJC, BMI, SPARCC, LEI) of psoriasis and control groups. Relationships between categorical variables were evaluated by chi-squared test. One-way analysis of variance (ANOVA) was used to compare quantitative data of three groups or more (eg, psoriasis clinical form, current treatment, sports activity). Correlations between quantitative variables (eg, disease duration and total score) were analysed using Spearman’s rank correlation coefficient. The study data were entered and analysed using Statistical Package for Social Sciences (SPSS) version 22 software. Throughout the statistical study, the significance level (p) was set at 0.05.

## RESULTS

### Clinical and US findings

A total of 40 patients with psoriasis and 40 sex- and age-matched controls were enrolled. The characteristics of the study population are shown in **Table 1**. Patients and controls were similar regarding their mean age, sex distribution, BMI, sports activity, and clinical findings (SJC, TJC, LEI, and SPARCC).

**Table 1.** Demographic, clinical data, and rheumatological findings in psoriasis group and controls.

**Parameters**	**Unit/category**	**Psoriasis group (n=40)**	**Control group (n=40)**	P-value^*^
**Age**	Years	51.9±15.90	52.3±14.83	0.905
**Sex**	Female	16 (40)	16 (40)	
				1
	Male	24 (60)	24 (60)	
**Smokers**	Yes	14 (35)	7 (17.5)	0.077
**BMI**	Kg/m^2^	27.14±5.55	27.21±4.78	0.506
**Diabetes mellitus**	Yes	6 (15)	8 (20)	0.562
**Hypertension**	Yes	13 (32.5)	7 (17.5)	0.124
**Hyperlipidaemia**	Yes	9 (22.5)	5 (12.5)	0.245
**Physical activity**	Inactive	8 (20)	26 (65)	
	Minimally Active	22 (55)	10 (25)	0.175
	Active	10 (25)	4 (10)	
**TJC**	>0	10 (25)	14 (35)	0.702
**TJC**		0 (0-1)	0 (0-1)	0.390
**SJC >0**	>0	2 (5)	1 (2.5)	0.816
**SJC**		0 (0-0)	0 (0-0)	0.320
**LEI**		0 (0-2)	1 (0-2)	0.804
**SPARCC**		1 (0-4)	2 (0-4)	0.872
**CRP**	mg/l	2.05 (0.07-7)	1 (0-2.95)	**0.037**

BMI: Body Mass Index; TJC: Tender joint count; SJC: swollen joint count; LEI: Leeds Enthesitis Index; SPRACC: Spondyloarthritis Research Consortium of Canada; CRP: C-reactive protein.

Quantitative data were mean±SD or median (IQR). Categorical data were number (%).

* p-values <0.05 (Student’s t-test or Mann-Whitney test or chi-squared test).

The mean disease duration was 16.86±16.04 years [1–57], the mean PASI score was 10.48±10.43 [0–44]. Clinical forms of psoriasis were: plaque psoriasis in 90% of cases, guttate psoriasis in 5% of cases, and pustular psoriasis in 5% of cases. Nail involvement was found in 14 patients (35%) and scalp psoriasis in 15 (37.5%). The mean NAPSI score was 6.08±8.85 [4–22]. The BSA indicated severe psoriasis in 17 patients (42.5%), moderate in 19 (47.5%), and mild in four patients (10%). Patients had a statistically significant higher CRP level than controls, but the value was low in both groups (median value 2.05 mg/l [0.07–7] versus 1mg/l [0–2.95], p= 0.037).

At inclusion, 24 (60%) patients were receiving topical steroids, 14 (35%) were receiving methotrexate (MTX) (mean dose 14.33±2.58mg/week [10–20] and mean duration 40.41±12.74 months [0.16–336]), four patients were receiving aciterin and six were on phototherapy. Among the 1040 scanned joints in each group, sub-clinical synovitis was found in seven joints in psoriasis patients (0.68%) (2 wrists, 5 MCP) and 3 joints in controls (0.29%) (3 MCP) (p= 0.420) (**Table 2**). No PDUS signal was found in the psoriasis group, whereas one MCP in controls showed power Doppler activity.

**Table 2.** Distribution of US findings in psoriatic patients and controls.

**Studied structures/US score**	**Psoriasis group**	**Controls**	P-value*
**Presence of US synovitis**			
**Total synovitis**			
Per patient	7/40 (17.5)	3/40 (7.5)	0.487
Per joint	7/1040 (0.68)	3/1040 (0.29)	0.420
**Wrist synovitis**			
GSUS	2 (5)	0 (0)	0.156
PDUS	0 (0)	0 (0)	NA
**MCP synovitis**			
GSUS	5 (12.5)	3 (7.5)	0.462
PDUS	0 (0)	1 (2.5)	0.320
**IPP synovitis**			
GSUS	0 (0)	0 (0)	NA
PSUS	0 (0)	0 (0)	NA
**Presence of US enthesitis**			
Per patient	37/40 (92.5)	16/40 (40)	<0.001
Per entheseal site	184/880 (20.90)	42/880 (4.78)	<0.001
**Abnormal entheses**			
Deep digital flexor	19/320 (5.94)	1/320 (1.25)	< 0.001
Common tensor tendon	26/80 (32.5)	10/80 (12.5)	0.011
Brachial triceps	8/80 (10)	3/80 (3.75)	0.145
Quadriceps	17/80 (21.25)	6/80 (7.5)	0.040
Proximal patellar tendon	20/80 (25)	3/80 (3.75)	0.001
Distal patellar tendon	33/80 (41.25)	4/80 (5)	<0.001
Calcaneal tendon	38/80 (47.5)	10/80 (12.5)	<0.001
Plantar fascia	23/80 (28.75)	7/80 (8.75)	0.007
**US scores**			
Inflammatory score	2.85 ±3.34	0.58 ±1.17	< 0.001
Damage score	3 ±2.57	0.60 ±1.41	< 0.001
Total score	5.85 ±5.20	1.18 ±2.07	< 0.001

US: ultrasonography, MCP: metacarpophalangeal, GSUS :Grayscale, PDUS: power Doppler, IPP: proximal interphalangeal, NA : not applicable.

Quantitative data were mean ±SD. Categorical data were number (%).

* p-values <0.05 (Student’s t-test or chi-squared test).

Almost 880 entheses had been scanned in each group. In psoriasis patients, the US showed at least one sign of enthesitis in 184 entheseal sites (20.90%) (**Table 2).** The calcaneal enthesis was the one with the highest number of enthesitis signs (47.5%), followed by distal patellar enthesis (41.25%), lateral epicondyle enthesis (32.5%), plantar aponeurosis enthesis (28.75%), the proximal patellar enthesis (25%), and the deep digital flexor enthesis (5.94%) (**Table 2**).

**Figure 2.**
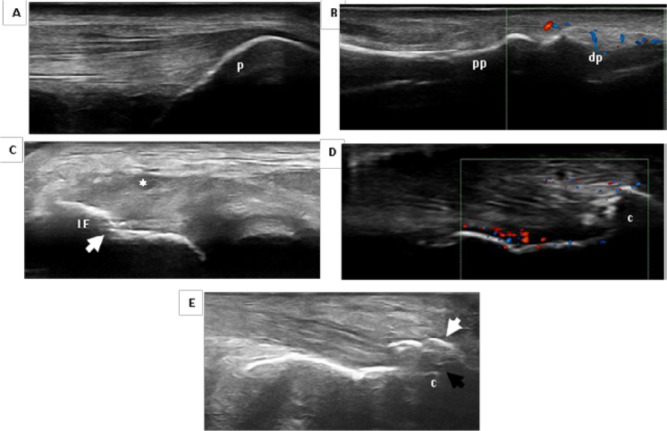
(A) Longitudinal ultrasonographic image of the quadriceps tendon shows a normal aspect of the enthesis. (B) Longitudinal ultrasonographic image of the deep flexor tendon of the 3rd finger shows power Doppler signal. (C) Longitudinal ultrasonographic image of the common extensor tendon insertion shows bone erosions (white arrow) abnormal thickening and hypoechogenicity (asterisk). (D) Longitudinal ultrasonographic image of the calcaneal tendon shows power Doppler signal. (E) Longitudinal ultrasonographic image of the calcaneal tendon shows enthesophyte (white arrow) and bone erosion (black arrow). p: patella ; md: medial phalange; dp: distal phalange; LE: lateral epicondyle; c: calcaneus.

The most frequent elementary lesion was entheseal thickening (n=70/880), followed by enthesophytes (n=60/880), bone erosion (n=54/880), enthesis hypoechogenicity (n=21/880), and enthesis calcification (n=10/en880) respectively. Different ultrasonographic pathological findings in our patients are illustrated in **Figure 2.**

In the control population, the US found at least one sign of enthesitis in 42 of the 880 (4.78%) entheseal sites (P<0.001). In this group, the entheseal site with the highest number of US signs of enthesitis was the calca-neal enthesis (10/80) (12.5%) and the lateral epicondyle enthesis (10/80) (12.5%).

The mean inflammatory score, damage score, and total score values were significantly higher in patients rather than in controls (P < 0.001 each) (**Table 2**).

### Correlation between US findings, clinical features, and disease characteristics of psoriasis patients

The age was fairly correlated with: the number of US enthesitis per patient (r=0.523, p=0.001), the inflammatory score, damage score, and total score (r=0.399, p=0.011; r=0.442, p=0.004; r=0.475, p=0.002 respectively).

No statistically significant association was found between US enthesis findings (number of enthesitis per patient, scores) and psoriasis duration, PASI, NAPSI, BMI, and sports activities. The BSA was correlated with the inflammatory score, but with no statistical significance (p=0.067). Scalp psoriasis was correlated with a higher number of US enthesitis per patient (p=0.020).

A statistically significant correlation was found between the number of US enthesitis per patient, and LEI and SPARCC scores (r=0.317; p=0.046, r=0.424; p=0.006, respectively). The SPARCC score was more correlated than LEI with inflammatory score, damage score, and total score (**Table 3**). Both LEI and SPARCC were correlated with the PDUS findings (r= 0.470; p=0.001, r= 0.437; p=0.002, respectively) (**Table 3**).

**Table 3.** Correlations of number of US enthesitis per patient and US scores and abnormalities with LEI and SPARCC.

	**LEI**		**SPARCC**	
	**r**	**p**	**r**	**p**
US enthesitis per patient	0.317	**0.046**	0.424	**0.006**
**US scores**				
Inflammatory score	0.239	0.138	0.407	**0.009**
Damage score	0.293	0.066	0.356	**0.024**
Total score	0.298	0.061	0.435	**0.005**
**US abnormality**				
Hypoechogenicity	0.194	0.115	0.357	**0.015**
Tendon-thickening	0.256	**0.055**	0.394	**0.006**
Enthesophytes	0.388	**0.007**	0.410	**0.004**
Bone erosion	0.071	0.331	0.184	0.127
Calcification	0.076	0.321	0.051	0.378
PDUS signal	0.470	**0.001**	0.437	**0.002**

LEI: Leeds Enthesitis Index, SPRACC: Spondyloarthritis Research Consortium of Canada, US: ultrasonography, PDUS: power Doppler. “Pearson r”:

a “fair” if it was between 0.30-0.50; and

b “weak or no association” if it was < 0.30.

Dealing with the influence of current treatment for psoriasis, there was no significant difference between the US scores of patients receiving MTX and those receiving other treatments (topical steroids, systemic retinoids). However, the dose of MTX was inversely correlated with the damage score (r=-0.445, p= 0.169), unlike the total duration of MTX intake, which was not correlated with the different US scores.

## DISCUSSION

Enthesitis is one of the hallmarks of Spondyloarthritis (SpA) including PsA.15 The high-resolution US combined with the Doppler technique has been studied over the past two decades to show its validity in revealing subclinical synovitis and enthesitis in inflammatory arthritis.^27,28^

In this study, we aimed to detect subclinical synovitis and enthesitis using the GSUS and PDUS scores in psoriasis patients compared with controls. We blindly investigated almost all relevant joints and entheses. We also studied the deep flexor tendon enthesis of the fingers which is rarely scanned in this context, and this is the major strength of our study. Our findings showed that US enthesitis and synovitis are more frequent in patients with psoriasis.

The US role in the detection of enthesitis has been demonstrated in patients with SpA first by D’Agostino.^29^ Lately, several US scores have been established for the assessment of US enthesitis in patients with PsA, interesting lower limb (GUESS),^30^ or both lower and upper limbs (MASEI).^25^ Currently, the GRAPPA has initiated a workgroup in order to develop a diagnostic US tool for enthesitis: the DUET.^31^

Our results were in accordance with those of previous studies that assessed entheseal US abnormalities in larger populations of psoriatic patients without musculoskeletal involvement [16;31-35]. According to the US score, the studies were interested in the enthesis of the lower limb or both lower and upper limbs. In our study, we assessed the enthesis of both the lower and upper limbs. Most studies had concluded that subclinical US enthesitis was more frequent in patients with psoriasis compared to controls by using different US scores (**Table 4**). The prevalence of US enthesitis in our patients (20.9% among the examined entheses) was in agreement with that of the literature (from 2.5% up to 67.8% within the examined entheses)^17,36-38^ (**Table 5**). This discrepancy in prevalence could be due to the different total number of entheses examined in each study. In fact, there is no consensus and some entheseal sites seem to have abnormalities in both PsA and psoriasis patients with no musculoskeletal symptoms.^39^

**Table 4.** Comparison between US enthesitis findings in the literature according to different scores.

**Study, yetar^ref^**	**Study populations Cases/Controls (Size)**	**Scanned entheses**	**US scores**	**p**
**Gisondi et al. 2007^31^**	Psoriasis (30) Other dermatosis (30)	QT,PPT,DP,CT, PPF	.GUESS score^a^ . Case :7.9± 0.6 . Controls :2.9± 0.3	<0.0001
**Eder et al. 2014^32^**	Psoriasis (66) Healthy controls (60)	TT QT PPT,DPT CT, PPF	.Modified MASEI score .Inflammatory score^b^ .Case:2 (4) .Controls: 1(2)	0.002
			.Damage score^b^ .Case:4 (6) .Controls: 0.023 (6)	0.1
			.Total score^b^ .Case:6 (8) . Controls : 3.5 (7.5)	0.02
**Hamdy et al. 2015^16^**	Psoriasis with rheumatic manifestations without fulfulling CAPSAR criteria (50) Healthy controls (20)	TT QT PPT,DPT CT, PPF	.MASEI score^a^ .Case:27.8±5.4 .Controls : 12.2±4.3	0.001
**Günaydın et al. 2020^33^**	Psoriasis (30) Healthy controls (30)	SST CET, CFT GT QT, PPT CT, PPF	.SPARCC US score^a^ .Case:4.7 ± 3.5 .Controls : 2.9 ± 2.3	0.04
**Vyas et al. 2020^35^**	Psoriasis (50) Healthy controls (50)	TT,QT, PPT,DPT, CT, PPF	.MASEI score^a^ .Case:12.72±7.55 .Controls : 5.14±4.69	0.000001
**Our study, 2021**	Psoriasis (40) Healthy controls (40)	DDF,CET, TT, QT, PPT,DPT, CT, PPF	.Inflammatory score^a^ .Case:2.85 ±3.34 .Controls :0.58 ±1.17	<0.001
			.Damage score^a^ . Case: 3 ±2.57 . Control: 0.60 ±1.41	<0.001
			.Total score ^a^ .Case:5.85 ±5.20 .Controls : 1.18 ±2.07	<0.001

US : ultrasonography, QT: quadriceps tendon, PPT: proximal patellar tendon, DPT: distal patellar tendon, CT: calcaneal tendon, PPF: proximal plantar fascia, TT: triceps tendon, SST: supraspinatus tendon, CET: common extensor tendon, CFT: common flexor tendon, GT: great trochanter, DDF: deep digital flexor.

a: (mean± SD),

b: (median(IQR))

**Table 5.** Comparison between US enthesitis prevalences in the literature according to the scanned entheses.

**Study, year^ref^**	**Study populations Cases/Controls (size)**	**Scanned entheses**	**US enthesitis n (%)**		**p**
			**Cases**	**Controls**	
**Naredo et al. 2011^17^**	Psoriasis (162) Healthy controls (60)	DDF PPT,DPT CT, PPF	285/2457 (11.6)	44/818 (5.3)	<0.0005
**Gutierrez et al. 2011^36^**	Psoriasis (45) Healthy controls (45)	QT PPT,DPT CT, PPF	148/450 (32.9)	38/450 (8.4)	<0.0001
**Freeston et al. 2012^37^**	Early PsA (42) Healthy controls (10)	CET DPT CT, PPF	24/296 (8.1)	9/78 (11.5)	NP
**Hamdy et al. 2015^16^**	Psoriasis with rheumatic manifestations without fulfulling CAPSAR criteria (50) Healthy controls (20)	TT QT PPT,DPT CT, PPF	407/600 (67.8)	20/240 (8.3)	0.004
**Elnady et al. 2019^38^**	Psoariasis (109) Healthy controls (90)	CET, CFT PPT,DPT CT, PPF	22/872 (2.5)	7/720(0.97)	0.01
**Our study 2021**	Psoariasis (40) Healthy controls (40)	DDF CET, TT QT PPT,DPT CT, PPF	184/880 (20.90)	42/880 (4.78)	<0.001

US: ultrasonography; DDF: deep digital flexor; PPT: proximal patellar tendon; DPT: distal patellar tendon; CT: calcaneal tendon; PPF: proximal plantar fascia; QT: quadriceps tendon; TT: triceps tendon; CET: common extensor tendon; CFT: common flexor tendon; NP: not precised.

We studied the entheseal sites that are often studied in the literature: lateral epicondyles, triceps tendon insertion into the olecranon tuberosity, proximal, and distal patellar tendons, calcaneal tendon, and plantar fascia. We added the deep flexor tendon of the finger enthesis, which is rarely studied by US to screen for enthesitis in psoriatic patients.

Naredo et al. had studied this enthesis in 136 patients with psoriasis and 46 healthy sex- and age-matched controls, but did not find a significant difference compared to controls (p=0.436).^17^ Our findings showed a statistically higher enthesitis rate at this site compared to controls (19/320 (5.94%) versus 1/320 (1.25%), p< 0.001).

The age of our patients was correlated with a higher number of US enthesitis in the psoriasis group (p=0.001). All US scores (inflammatory, damage, total) increased with age (p=0.011; p=0.004; p=0.002, respectively). Inconsistent with our results, Gisondi et al. and Eder et al. found a positive correlation between the age and the US scores (GUESS and MASEI).^31,32^ Bakirci et al., however, confirmed this correlation in healthy subjects.^40^

The patient gender was not correlated with the number of US enthesitis nor with the different US scores in both psoriasis and control groups, which was confirmed by Hamdy et al. in a follow-up case-control US study using MASEI score.^16^ However, male sex was a risk factor for the development of enthesitis (p = 0.003) in a study of healthy participants.^40^

The duration of psoriasis in our study ranged from one year to 57 years and it was not correlated with the number of US enthesitis nor to different US scores. In a literature review, which focused on the predictive factors of PsA, the duration of psoriasis was not correlated with a higher incidence of PsA,^41^ unlike Wilson et al.,42 who confirmed that the cumulative incidence of PsA increased with the disease duration.

Three psoriasis phenotypes were found among our population: plaque psoriasis (90%), guttate psoriasis (5%), and pustular psoriasis (5%). Several authors advanced the hypothesis of a psoriasis risk phenotype for the development of PsA such as nail and scalp involvement.^6,41–42^ This is consistent with our results. In fact, a greater number of US enthesitis were found in patients with scalp involvement (p=0.020), but no association was observed for the other psoriasis phenotypes.

Savage et al. in their literature review advanced the absence of a link between the severity of psoriasis (PASI) and the likelihood of developing PsA.^6^ That was confirmed in later studies.^16,33,43^ Wilson et al. contrari-wise, showed that severe psoriasis were a predictive factor of developing PsA.^44^ Actually, it has been shown that synovium and skin lesions in PsA express the proinflammatory cytokine tumor necrosis factor α (TNFα); therefore, the larger affected skin area of psoriasis may result in increased systemic levels of TNFα and therefore in developing PsA.^45,46^

Mechanical stress, such as overweight and physical activity, could be risk factors and induce structural changes (enthesophyte, calcification, erosion) of the enthesis, especially in the lower limbs.^31,32,40,47^ According to enthe-seal sites, different findings are reported. In the study of Gisondi et al. investigating the lower limb and using the GUESS score, the authors identified a link between BMI and US enthesitis.^31^ However, Eder et al. by studying both upper and lower limbs using the MASEI score, did not observe a significant difference between US findings of patients (psoriasis patients and healthy controls) and BMI over than 30. Whereas, that was found in patients with a BMI less than 30.^32^ In our population, we did not observe associations between US enthesitis and BMI or physical activity intensity. However, for US scores, sedentary patients (inactive) had higher inflammation score values compared to minimally active or active patients (p=0.055). Studies results are controversial about the link between physical activity and US enthesis abnormalities, some authors support this notion,^35^ while others confirm that regular physical activity could improve US outcomes.^48^

In our study, we sought the influence of disease-modifying treatment on US entheseal abnormalities. A higher dose of MTX reduced the damage score, but not significantly (r=-0.445, p=0.169). Few studies have investigated the influence of MTX intake on the structural and inflammatory changes of enthesis. Acquacalda et al. provided US monitoring of the enthesis of two groups of patients (psoriasis, PsA) treated with methotrexate alone or in combination with biologic drugs.^49^ An improvement in the US abnormalities of the enthesis was noted for the psoriasis group after six months of treatment (p=0.021), however, there was no significant improvement for the PsA group (p=0.164). These results suggest the role of systemic disease-modifying treatments for psoriasis on the subclinical evolution of enthesitis and, by analogy, the evolution towards a possible PsA in the absence of treatment or its optimisation.^49^

Our study has some limitations: first, the sample size of our study was the major limitation and does not allow us to generalise our results. Second, axial joint and entheseal involvement, which are commonly seen with PsA, were not included in this study since it cannot be properly imaged by US. Third, although the US examination was carried out by an expert; ultrasonography remains an operator-dependent examination. Finally, more than a third of patients were on MTX which could alter US findings, but we noted no significant difference between the US scores of patients receiving (MTX) and those receiving other treatments. The results should be interpreted with caution. A follow-up of psoriatic patients with US abnormalities should be conducted in order to detect eventually early features of the disease and to establish the incidence of PsA and other risk factors.

## CONCLUSION

In conclusion, our results suggest that psoriasis is associated with a higher prevalence of asymptomatic US synovitis and enthesitis. Future longitudinal studies with larger sample sizes should consider whether US findings in asymptomatic psoriasis patients have predictive value in developing PsA. US could be used as a screening tool to detect early PsA, especially in patients with scalp involvement and a positive SPARCC score. Such patients should be referred to a rheumatologist in an early stage, seeking a better outcome.

## Data Availability

All data underlying the results are available as part of the article and no additional source data are required. All data are available in our rheumatology department and could be consulted at any time.
